# The effects of dietary macronutrient composition on lipid metabolism-associated factor gene expression in the adipose tissue of chickens are influenced by fasting and refeeding

**DOI:** 10.1186/s40608-017-0150-8

**Published:** 2017-05-10

**Authors:** Guoqing Wang, Betty R. McConn, Dongmin Liu, Mark A. Cline, Elizabeth R. Gilbert

**Affiliations:** 1 Department of Animal and Poultry Sciences Virginia Polytechnic Institute and State University, Blacksburg, VA 24061 USA; 2 Department of Human Nutrition, Foods and Exercise Virginia Polytechnic Institute and State University, Blacksburg, VA 24061 USA

**Keywords:** Dietary macronutrients, Fasting, Adipose tissue, mRNA abundance, Chicks

## Abstract

**Background:**

Broiler chickens are compulsive feeders that become obese as juveniles and are thus a unique model for metabolic disorders in humans. However, little is known about the relationship between dietary composition, fasting and refeeding and adipose tissue physiology in chicks. Our objective was to determine how dietary macronutrient composition and fasting and refeeding affect chick adipose physiology during the early post-hatch period.

**Methods:**

Chicks were fed one of three isocaloric diets after hatch: high-carbohydrate (HC; control), high-fat (HF; 30% of ME from soybean oil) or high-protein (HP; 25% vs. 22% crude protein). At 4 days post-hatch, chicks were fed (continuous ad libitum access to food), fasted (3 h food withdrawal), or refed (fasted for 3 h and refed for 1 h). Subcutaneous, clavicular, and abdominal adipose tissue was collected for histological analysis and to measure gene expression, and plasma to measure non-esterified fatty acid (NEFA) concentrations (*n* = 6–10 per group).

**Results:**

Adipose tissue weights were reduced in chicks that were fed the HP diet and adipocyte diameter was greater in the adipose tissue of chicks that ate the HF diet. Consumption of diets differing in protein and fat content also affected gene expression; mRNAs encoding fatty acid binding protein 4 and a lipolytic enzyme, monoglyceride lipase, were greater in chicks fed the HC and HF than HP diet in all three adipose tissue depots. Fasting influenced gene expression in a depot-dependent manner, where most fasting and refeeding-induced changes were observed in the clavicular fat of chicks that consumed the HC diet. Fasting increased plasma NEFA concentrations in chicks fed the HC and HP diets.

**Conclusions:**

The decreased adipose tissue deposition in chicks fed the HP diet is likely explained by decreased rates of adipogenesis. Consumption of the HF diet was associated with greater adipose tissue deposition and larger adipocytes, likely as a result of greater rates of adipocyte hypertrophy. The depot-dependent effects of diet and fasting on gene expression may help explain mechanisms underlying metabolic distinctions among subcutaneous and visceral fat depots in humans.

## Background

Intensive selection for growth rate and meat yield in broiler chickens has led to correlated increases in voluntary food consumption, fat deposition and incidence of metabolic disorders in breeders [1]. Thus, chickens may serve as a model to better understand the genetic and molecular basis for metabolic disorders in humans. In mammals, it is well-known that subcutaneous fat can prevent other tissues from accumulating excessive lipids that can cause lipotoxicity, therefore acting as a buffer for the daily incorporation of dietary fat [2]. It is also considered to be metabolically benign as compared to other anatomical depots. However, visceral fat (body fat stored within the abdominal cavity) is associated with metabolic disorders [3], yet little is known about whether such differences exist in avian species.

Dietary macronutrient composition not only affects appetite in birds and mammals but also regulates adipose tissue physiology. When mice are fed a high-fat (HF) diet, hypertrophy occurs in visceral fat whereas hyperplasia dominates in subcutaneous fat as both depots expand in response to excess caloric intake [4]. However, little is known about the effects of dietary macronutrient composition on adipose tissue development in avian species. We recently reported that a HF diet enhanced the sensitivity to the effects of exogenous neuropeptide Y (NPY) on food intake in chicks and that NPY in turn led to increased food intake in chicks that consumed a high-protein (HP) and high-carbohydrate (HC), but not HF diet [5].

In response to food deprivation and refeeding, a complex array of adaptive metabolic changes are triggered that mediate dynamic alterations in appetite regulation, energy storage and expenditure in peripheral tissues in response to the changes in energy availability. The effects of fasting and refeeding on the gene expression of metabolism-associated genes in different tissues has been extensively studied in both birds and mammals, however, there is little known about the effects of diet on molecular changes in the adipose tissue of chicks. Therefore, the purpose of this study was to evaluate effects of dietary macronutrient composition and fasting and refeeding on adipose tissue physiology in broilers chicks during the first 4 days post-hatch.

## Methods

### Animals

The Institutional Animal Care and Use Committee at Virginia Tech reviewed and approved all animal protocols and chicks were cared for in accordance with the Guide for the Care and Use of Laboratory Animals. Cobb-500 broiler chicks were transported from a local hatchery on the morning of hatch and were housed in electrically heated and thermostatically controlled cages. The temperature was 30 ± 2 °C with 50 ± 5% relative humidity and 24 h of light. Chicks were randomly assigned to receive one of three diets, with free access to food and water. Diets were formulated to be isocaloric (3,000 kcal/kg) as shown in Table 1 and mixed at Augusta Cooperative Feed Mill (Staunton, Virginia, USA). The HP diet was formulated to contain 25% crude protein and the HF diet to have 30% of the ME derived from calories in soybean oil. Crude protein and fat values were experimentally verified for all diets (Table 1).

**Table 1:** Ingredient and chemical composition of experimental chick diets

Ingredient (% as fed)	High-carbohydrate	High-protein	High-fat
Corn grain	60.37	43.61	31.28
Soybean meal	33.99	48.80	36.94
Soy hulls	0.00	0.00	17.27
Dicalcium phosphate	1.50	1.42	1.56
Limestone	1.21	1.14	0.89
Soybean oil	1.13	3.65	10.20
Vitamin/mineral premix^a^	1.00	1.00	1.00
Methionine 99%	0.26	0.12	0.32
L-Lysine HCL 78%	0.19	0.00	0.16
Sodium bicarbonate	0.15	0.15	0.15
L-Threonine	0.09	0.00	0.12
Coban 90^b^	0.05	0.05	0.05
Choline-Cl 60%	0.05	0.05	0.05
Phytase-Ronozyme^c^ 10000^d^	0.01	0.01	0.01
Kcal ME/kg	3,000	3,000	3,000
Crude protein (%)^d^	22	25	22
Crude fat (%)^d^	3.0	4.6	10.2
Crude fiber (%)	1.8	8.3	1.8

^a^Guaranteed analysis (per kg of premix): Manganese, 25.6 g; selenium, 120 mg; zinc, 30 g; Vitamin A, 4409, 171.076 IU; Vitamin D3, 1410,934.744 ICU; 13,227.513 IU; D-biotin, 88.183 mg

^b^Coban 90 (Elanco Animal Health) contains 90 g of Monensin sodium per pound of premix and is included in the diet as a coccidiostat

^c^DSM Nutritional Products, Ltd

^d^Analyzed at Experiment Station Chemical Laboratories at University of Missouri

At 4 days post-hatch, chicks from each diet were randomly assigned to one of three treatments: fasting (3 h), refeeding (1 h of ad libitum access to food after 3 h of fasting) and feeding (continuous ad libitum access to food), with *n* = 10 chicks per group. Chicks were euthanized, sexed by visual inspection of gonads, and tissues collected as described below.

### Adipose tissue depot weights and histology

Subcutaneous, clavicular, and abdominal adipose tissue samples were collected from *n* = 10 chicks as described [6, 7], from each of the three dietary groups (HC, HF, and HP) at 4 days post-hatch (all continuously fed). Adipose tissue depots were weighed and values converted into a percentage of the chick’s body weight. Samples were prepared for histological evaluation as described [6]. Samples were washed in ice-cold phosphate-buffered saline, submerged in neutral-buffered formalin and rocked on a platform overnight at 4 °C. Samples were then dehydrated in a graded ethanol series, embedded in paraffin blocks, sectioned at 5 μm on a microtome, and mounted on glass slides. One section was mounted per slide with two slides (at least 200 μm apart) per sample. Only samples from chicks assigned to the “fed” treatment within each dietary group were used for histology. Hematoxylin and eosin staining was performed and images were captured with a Nikon Eclipse 80i microscope and DS-Ri1 color camera. The digital images were then analyzed as described [6] using NIS-Elements Advanced Research Software (Nikon). The density (threshold method), diameter, and area of all adipocytes within the field of an image were determined. Adipocytes were evaluated as binary objects with the restriction that measurements must exceed 100 μm^2^. Size distributions in each image were also analyzed.

### Plasma NEFA concentrations

Approximately 200 μL of blood was collected from the trunk (*n* = 10 chicks) via capillary blood collection tubes (Microvette®) immediately following euthanasia and decapitation. After collection, samples were centrifuged at 2,000 x g at room temperature and plasma isolated. Plasma NEFA concentrations were measured using the NEFA-HR2 kit (Wako Diagnostics) according to the manufacturer’s instructions. Absorbance was measured at 550 nm using an Infinite M200 Pro multi-mode plate reader (Tecan). Sample concentration was calculated using the following formula: Sample Concentration = Standard Concentration × (Sample Absorbance)/(Standard Absorbance). Units for the concentrations are reported as mEq/L.

### Total RNA isolation and real-time PCR

Adipose tissue samples were collected (*n* = 10 per group) and submerged in RNAlater (Qiagen) and processed for gene expression analyses as described [6]. Samples were disrupted in 1 mL Isol RNA Lysis reagent (5-Prime, Gaithersburg, MD, USA) with 5 mm stainless steel beads (Qiagen, Valencia, CA, USA) and a Tissue Lyser II (Qiagen) for 2 × 2 min at 25 Hz. Following the step of addition to 100% ethanol, samples were applied to spin columns and total RNA isolated using Direct-zol RNA Kits (Zymo Research) with the on-column RNase-free DNase I (Zymo Research) treatment. Total RNA integrity was verified by agarose-formaldehyde gel electrophoresis and concentration quantified and purity assessed by spectrophotometry at 260/280/230 nm with a Nanophotometer Pearl (IMPLEN, Westlake Village, CA, USA). Reverse transcription was performed as described [8] with a High Capacity cDNA Reverse Transcription Kit (Applied Biosystems) and 200 ng of total RNA, following the manufacturer’s instructions under the following conditions: 25 °C for 10 min, 37 °C for 120 min and 85 °C for 5 min. Real time PCR primers were designed with Primer Express 3.0 software (Applied Biosystems) and the amplification efficiency (within 5% of reference gene) was validated for all primer pairs (Table 2). Duplicate real-time PCR reactions contained 5 μl Fast SYBR Green Master Mix (Applied Biosystems), 0.25 μL each of 5 μM forward and reverse primers, 1.5 μL of nuclease-free water, and 3 μl of 10-fold diluted cDNA. A 7500 Fast Real-Time PCR System (Applied Biosystems) was used under the following conditions: 95 °C for 20 s and 40 cycles of 90 °C for 3 s plus 60 °C for 30 s. A melting curve analysis, consisting of 95 °C for 15 s, 60 °C for 1 min, 95 °C for 15 s and 60 °C for 15 s, was performed after each PCR to validate amplicon specificity.

**Table 2:** Primers used for real time PCR

Gene^a^	Primers sequence (5′–3′); Forward/Reverse	Accession No.
*β-actin*	GTCCACCGCAAATGCTTCTAA/TGCGCATTTATGGGTTTTGTT	NM_205518.1
*PPARγ*	CACTGCAGGAACAGAACAAAGAA/TCCACAGAGCGAAACTGACATC	NM_001001460.1
*C/EBPα*	CGCGGCAAATCCAAAAAG/GGCGCACGCGGTACTC	NM_001031459.1
*C/EBPβ*	GCCGCCCGCCTTTAAA/CCAAACAGTCCGCCTCGTAA	NM_205253.2
*FABP4*	CAGAAGTGGGATGGCAAAGAG/CCAGCAGGTTCCCATCCA	NM_204290.1
*SREBP1*	CATCCATCAACGACAAGATCGT/CTCAGGATCGCCGACTTGTT	NM_204126.1
*KLF7*	GATGCTGGTTTTCCTCACAGTTT/CCTCCTGTCCCAAAAGTGTTCA	XM_004942644.2
*GATA2*	CCACGAAGCAAGGCCAGAT/GGTAGCGGTTGCTCCACAGT	XM_015293080.1
*AGPAT9*	CCCATAGATGCGATCATTTTGA/CGTGAACTTGGCCAACCAT	NM_001031145.1
*MGLL*	GCAGACGAGCATAGACTCA/GGGAATAGCCTGGTTTACAA	XM_015293082.1
*ATGL*	GCCTCTGCGTAGGCCATGT/GCAGCCGGCGAAGGA	NM_001113291.1
*CGI-58*	CGCCCAGTGGTGAAAC/GCCTTTTTGCCCATCCATAA	NM_001278145.1
*PLN1*	GGAGGACGTGGCATGATGAC/GGCCCTTCCATTCTGCAA	NM_001127439.1
*ACADL*	GACATCGGCACTCGGAAGA/CCTGGTGCTCTCCCTGAAGA	NM_001006511.2
*LPL*	GACAGCTTGGCACAGTGCAA/CACCCATGGATCACCACAAA	NM_205282.1
*NPY*	CATGCAGGGCACCATGAG/CAGCGACAAGGCGAAAGTC	M87294.1
*NPYR2*	TGCCTACACCCGCATATGG/GTTCCCTGCCCCAGGACT﻿A	NM_001031128.1

^a^Gene abbreviations: Peroxisome proliferator-activated receptor gamma: *PPARγ*; CCAAT/enhancer-binding protein alpha and beta: *C/EBPα* and *C/EBPβ*, respectively; Fatty acid binding protein 4: *FABP4*; Sterol regulatory element-binding transcription factor 1: *SREBP1*; Kruppel-Like Factor 7: *KLF7*; GATA binding protein 2: *GATA2*; 1-acylglycerol-3-phosphate O-acyltransferase 9: *AGPAT9*; Monoglyceride lipase: *MGLL*; Adipose triglyceride lipase: *ATGL*; Comparative gene identification-58: *CGI-58*; Perilipin 1: *PLN1*; Acyl-CoA dehydrogenase, long chain: *ACADL*; Lipoprotein lipase: *LPL*; Neuropeptide Y: *NPY*; *NPY* receptor 2: *NPYR2*

### Statistical analysis

The real time PCR data were analyzed using the ΔΔCT method, where ΔCT = CT target gene − CT actin, and ΔΔCT = ΔCT target sample − ΔCT calibrator [9]. The average of clavicular fat from chicks fed the HC diet that received the “fed” treatment was used as the calibrator sample. The fold difference (relative quantity; RQ) was calculated as 2^-ΔΔCT^. Analysis of variance (ANOVA) was performed for adipose depot weight, percent weights, adipocyte area and diameter, NEFA concentrations, and RQ values using the Fit Model platform of JMP Pro11 (SAS Institute, Cary, NC). Because sex and interactions between sex and diet did not significantly influence any of the traits measured in this study, sex was excluded from the statistical models. For weights and morphometric measurements, the statistical model included the main effects of diet, adipose tissue depot, and their interaction. Post hoc pairwise comparisons were carried out with Tukey’s test. For RQ data (analyzed within depot) and NEFAs, the model included the main effects of diet and fasting treatment and the interaction between them. The interaction on NEFAs was separated with Tukey’s test. Significant interactions from real time PCR data were separated with the Slice function of JMP and effects sliced within dietary group for each gene. Significant dietary effects were further analyzed by second ANOVAs with Tukey’s test to separate the means. All data are presented as means ± SEM. Differences were considered significant at *P* < 0.05.

## Results

### Adipose tissue depot weights and histology

At day 4 post-hatch, body weights were not different among groups and chicks fed HC and HF diets had greater fat depot weights (*P* = 0.0001) than chicks fed the HP diet (Table 3), even when expressed as a percentage of body weight (*P* = 0.0002). The subcutaneous fat was heaviest, clavicular fat intermediate, and abdominal fat the lightest (*P* = 0.0001). This was also observed when depots were expressed as a percentage of body weight (*P* = 0.0001). Adipocyte diameter was greater (*P* < 0.05) in chicks fed the HF diet than in chicks fed the HC or HP diet (Table 4). Adipocyte area and diameter were greatest in subcutaneous fat, intermediate in clavicular and smallest in abdominal fat (*P* < 0.0001).

**Table 3:** Adipose tissue depot weights

Effect^1^	Weights (g)	Weights (% BW)
Diet		
HC	0.33^a^	0.41^a^
HF	0.34^a^	0.42^a^
HP	0.23^b^	0.30^b^
SEM	0.017	0.021
*P*-value	0.0001	0.0002
Depot		
Subcutaneous	0.52^a^	0.65^a^
Clavicular	0.27^b^	0.34^b^
Abdominal	0.11^c^	0.14^c^
SEM	0.017	0.021
*P*-value	0.0001	0.0001
D × D	0.08	0.20

^1^Effects of diet (high-carbohydrate: HC; high-fat: HF; and high-protein: HP), adipose tissue depot (subcutaneous, clavicular and abdominal), and the interaction between diet (D) and depot (D) on adipose tissue weights and weights as a percentage of body weight. Values represent least squares means and pooled standard errors of the means with associated *P*-values for each effect (*n* = 10). Unique superscipts within an effect are significantly different at *P* < 0.05, Tukey’s test

**Table 4:** Adipocyte area and diameter in different adipose tissue depots

Effect^1^	Area (μm)	Diameter (μm)
Diet		
HC	368.5	20.3^b^
HF	442.4	22.4^a^
HP	361.4	20.3^b^
SEM	25.1	0.6
*P*-value	0.056	0.02
Depot		
Subcutaneous	526.0^a^	24.6^a^
Clavicular	419.1^b^	22.0^b^
Abdominal	227.1^c^	16.4^c^
SEM	25.1	0.6
*P*-value	0.0001	0.0001
D × D	0.96	0.97

^1^Main effects of diet (high-carbohydrate: HC; high-fat: HF; and high-protein: HP), adipose tissue depot (subcutaneous, clavicular and abdominal), and the interaction between diet (D) and depot (D) on adipocyte area and diameter. Images were analyzed using NIS-Elements Advanced Research Software (Nikon). The density, diameter, and area of all adipocytes within the field of an image were measured. Adipocytes were treated as binary objects with the restriction that measurements must exceed 100 μm^2^. Values represent least squares means and pooled standard errors of the means with associated *P*-values for each effect (HC: *n* = 7; HF: *n* = 6; HP: *n* = 7). Different superscipts within an effect are significantly different at *P* < 0.05, Tukey’s test

**Fig. 1:**
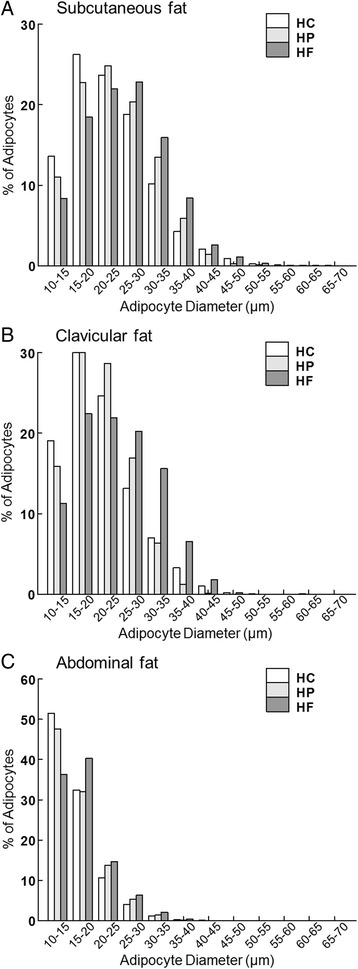
Adipocyte size distribution in (**a**) subcutaneous, (**b**) clavicular, and (**c**) abdominal adipose tissue at day 4 post-hatch in chicks fed a high-carbohydrate (HC), high-protein (HP) or high-fat (HF) diet. Images were captured with a Nikon Eclipse 80i microscope and DS-Ri1 color camera and analyzed using NIS-Elements Advanced Research Software (Nikon). The density, diameter, and area of all adipocytes within the field of an image were measured under 20x magnification. The threshold method was used to count adipocytes. *n* = 7 (HC), 6 (HF) and 7 (HP)

**Fig. 2:**
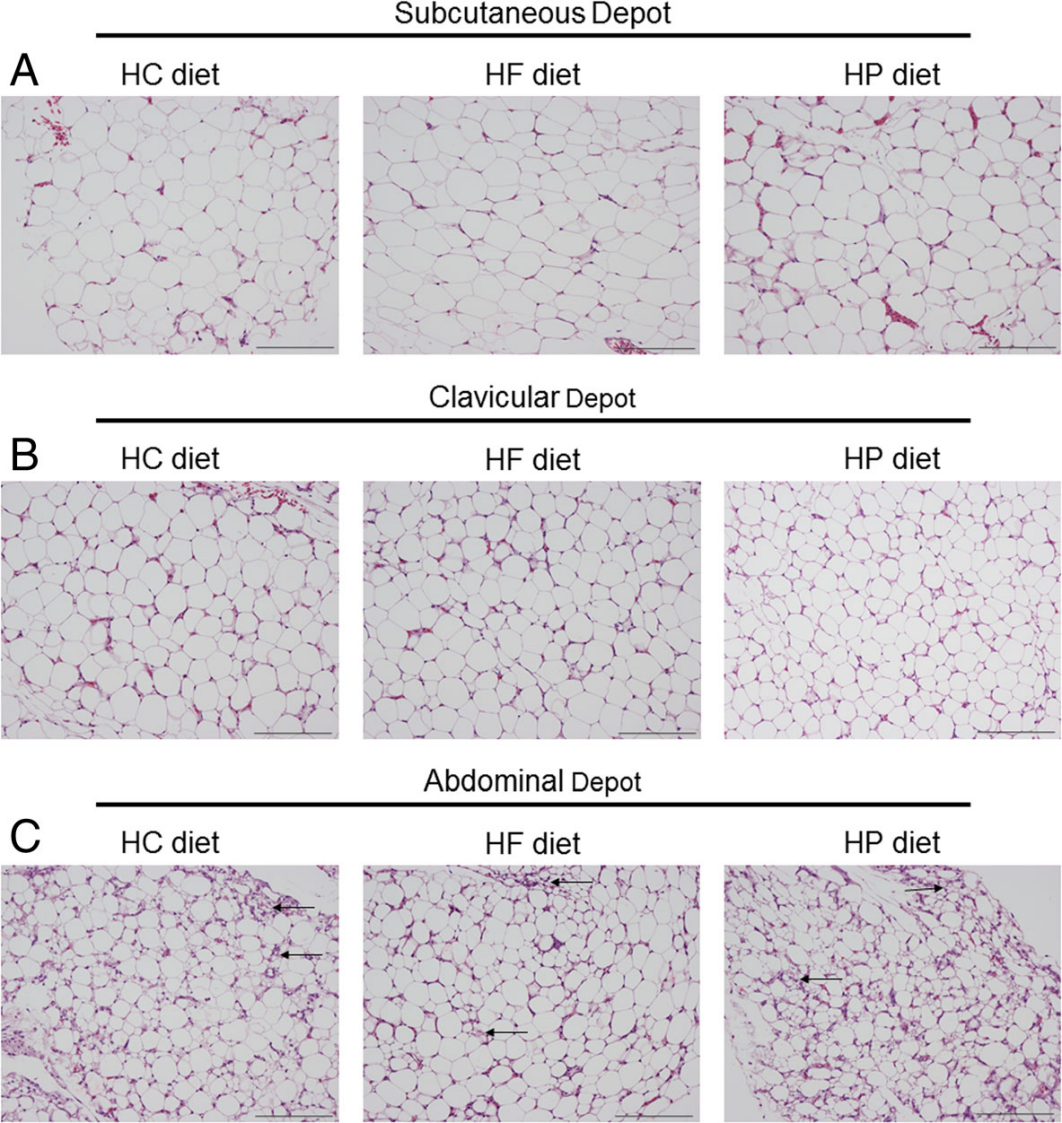
Adipose tissue histology at day 4 post-hatch in (**a**) subcutaneous, (**b**) clavicular, and (**c**) abdominal depots. Scale bar = 100 μm. Representative images of hematoxylin and eosin-stained sections from *n* = 7 (high-carbohydrate diet; HC), 6 (high-fat diet; HF) and 7 (high-protein diet; HP). Images were captured with a Nikon Eclipse 80i microscope and DS-Ri1 color camera

More than 64% and 59% of adipocytes were 10 to 25 μm in mean diameter in the subcutaneous fat of chicks fed the HC and HP diets, respectively, while only 49% of the adipocytes were 10 to 25 μm in the subcutaneous fat of chicks fed the HF diet (Fig. 1a). Most of the adipocytes (>75%) were 10 to 25 μm in mean diameter in the clavicular fat of chicks fed the HC and HP diets (Fig. 1b). However, only 55% of the adipocytes were smaller than 25 μm in mean diameter in the clavicular fat of chicks fed the HF diet. In the abdominal fat, most of the adipocytes (>91%) were 10 to 25 μm in mean diameter at day 4, irrespective of the diet (Fig. 1c). The gross histology (Fig. 2) also suggests that there were many highly-vascularized clusters of what might be preadipocytes in the abdominal fat (Fig. 2c), but not in the other two adipose depots of all chicks, independent of diet.

### Plasma NEFA concentrations

**Fig. 3:**
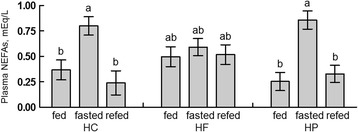
Interaction of diet and treatment on plasma non-esterified fatty acid (NEFA) concentrations. At day 4 post-hatch, chicks fed one of three diets (HC: high-carbohydrate; HF: high-fat; HP: high-protein) were either continuously fed (fed), fasted for 3 h (fasted) or fasted and refed for 1 h (refed) with *n* =10 per group. Values represent least squares means ± SEM. Different letters indicate a significant difference at *P* < 0.05; Tukey’s test

There was an interaction of feeding treatment and diet on plasma NEFA concentrations (*P* = 0.02). Plasma NEFAs were greater in fasted than fed or refed chicks that consumed the HC and HP diets (*P* = 0.02; Fig. 3). Plasma NEFAs were not affected by fasting or refeeding in chicks that consumed the HF diet. Plasma NEFAs were not different among dietary groups.

### mRNA abundance in subcutaneous adipose tissue

**Fig. 4:**
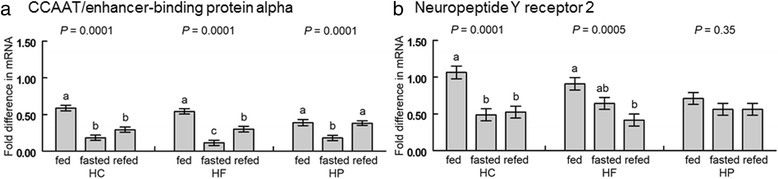
Interactions of diet and treatment on mRNA abundance of **a**) CCAAT/enhancer-binding protein alpha and **b**) Neuropeptide Y receptor 2 in subcutaneous fat. Chicks fed one of three diets (HC: high-carbohydrate; HF: high-fat; HP: high-protein) were either continuously fed (fed), fasted for 3 h (fasted) or fasted and refed for 1 h (refed) with *n* =10 per group. Values represent least squares means ± SEM. The *P*-values for the main effect of treatment are displayed above the bars for each dietary group. Different letters within a dietary group indicate a significant difference at *P* < 0.05; Tukey’s test

The mRNA abundance results for subcutaneous, clavicular, and abdominal adipose tissue are summarized in Tables 5, 6, and 7, respectively. Significant two-way interactions are displayed graphically. There were interactions of diet and feeding treatment on CCAAT/enhancer-binding protein alpha (*C/EBPα*) and NPY receptor 2 (*NPYR2*) in subcutaneous adipose tissue (Fig. 4). *C/EBPα* expression was greater in fed than fasted or refed chicks that consumed the HC diet, and greater in fed and refed than fasted chicks that consumed the HP diet, whereas in chicks that consumed the HF diet, expression was greatest in fed, intermediate in refed, and lowest in fasted chicks (*P* < 0.05; Fig. 4a). Expression of *NPYR2* was greater in fed than fasted or refed chicks that consumed the HC diet, greater in fed than refed chicks that consumed the HF diet, and was similar across treatments in chicks that consumed the HP diet (*P* < 0.05; Fig. 4b).

**Table 5:** Subcutaneous fat mRNA abundance at 4 days post-hatch

Effect^1^	*AGPAT9*	*FABP4*	*C/EBPα*	*C/EBPβ*	*KLF7*	*PPARγ*	*MGLL*	*SREBP1*
Diet								
HC	0.44	1.06^a^	0.35	1.77	1.31	0.45	2.60	0.51
HF	0.36	1.00^ab^	0.32	1.40	1.32	0.50	2.21	0.43
HP	0.36	0.84^b^	0.32	1.33	1.19	0.39	2.31	0.41
SEM	0.04	0.06	0.02	0.18	0.09	0.04	0.27	0.04
*P*-value	0.23	0.02	0.37	0.21	0.66	0.27	0.55	0.10
Fed	0.57^a^	1.03	0.51^a^	1.00^b^	1.28^b^	0.73^a^	2.42	0.67^a^
Fasted	0.31^b^	0.96	0.16^c^	2.26^a^	1.59^a^	0.32^b^	2.54	0.39^b^
Refed	0.31^b^	0.91	0.33^b^	1.18^b^	0.97^c^	0.32^b^	2.18	0.33^b^
SEM	0.04	0.05	0.02	0.17	0.09	0.04	0.26	0.04
*P*-value	0.0001	0.35	0.0001	0.0001	0.0001	0.0001	0.57	0.0001
D × T	0.13	0.22	0.003	0.31	0.77	0.21	0.67	0.06
Effect^1^	*GATA2*	*ATGL*	*PLN1*	*CGI-58*	*ACADL*	*NPY*	*NPYR2*	*LPL*
Diet								
HC	0.97	1.41	0.89	1.24	0.70	0.91	0.69	1.21
HF	1.05	1.42	0.77	0.70	0.56	0.64	0.66	1.41
HP	0.86	1.36	0.87	0.96	0.49	1.09	0.61	1.64
SEM	0.07	0.17	0.13	0.19	0.15	0.26	0.05	0.19
*P*-value	0.11	0.96	0.76	0.15	0.57	0.46	0.48	0.30
Treatment								
Fed	1.10	1.50	0.79	1.20	0.72	1.35	0.89^a^	1.55
Fasted	0.90	1.38	1.00	0.93	0.76	0.85	0.56^b^	1.25
Refed	0.89	1.31	0.74	0.77	0.28	0.44	0.50^b^	1.47
SEM	0.06	0.17	0.13	0.19	0.15	0.26	0.05	0.19
*P*-value	0.08	0.76	0.30	0.29	0.03	0.07	0.0001	0.50
D × T	0.26	0.76	0.56	0.51	0.14	0.76	0.03	0.37

^1^Values represent least squares means and pooled standard errors of the means with associated *P*-values for each effect. D × T: interaction between diet (D) and treatment (T). Different superscipts within an effect for each gene are significantly different at *P* < 0.05, Tukey’s test. Abbreviations: 1-acylglycerol-3-phosphate O-acyltransferase 9: *AGPAT9*; Fatty acid binding protein 4: *FABP4*; CCAAT/enhancer-binding protein alpha and beta: *C/EBPα* and *C/EBPβ*, respectively; Krüppel-like factor 7: *KLF7*; Peroxisome proliferator-activated receptor gamma: *PPARγ*; Monoglyceride lipase: *MGLL*; Sterol regulatory element-binding transcription factor 1: *SREBP1*; GATA-binding protein 2: *GATA2*; Adipose triglyceride lipase: *ATGL*; Perilipin 1: *PLN1*; Comparative gene identification-58: *CGI-58*; Acyl-CoA dehydrogenase, long chain: *ACADL*; Neuropeptide Y: *NPY*; NPY receptor 2: *NPYR2;* Lipoprotein lipase: *LPL*

**Table 6:** Clavicular fat mRNA abundance at 4 days post-hatch

Effect^1^	*AGPAT9*	*FABP4*	*C/EBPα*	*C/EBPβ*	*KLF7*	*PPARγ*	*MGLL*	*SREBP1*
Diet								
HC	0.62	0.90^a^	0.57	2.08	1.02	0.54	1.34^a^	0.69^a^
HF	0.48	1.09^a^	0.78	2.12	1.01	0.50	1.40^a^	0.60^ab^
HP	0.51	0.68^b^	0.50	1.30	1.09	0.44	0.98^b^	0.33^c^
SEM	0.09	0.06	0.15	0.33	0.07	0.05	0.12	0.07
*P*-value	0.34	0.0001	0.41	0.18	0.66	0.19	0.03	0.0008
Treatment								
Fed	0.77^a^	0.97^a^	0.84	1.12^b^	0.99^b^	0.78^a^	1.25	0.82^a^
Fasted	0.51^ab^	0.93^ab^	0.58	2.68^a^	1.33^a^	0.39^b^	1.13	0.41^b^
Refed	0.37^b^	0.76^b^	0.45	1.66^ab^	0.81^b^	0.33^b^	1.32	0.40^b^
SEM	0.08	0.06	0.15	0.32	0.07	0.05	0.12	0.07
*P*-value	0.006	0.04	0.19	0.007	0.0001	0.0001	0.51	0.0001
D × T	0.01	0.87	0.19	0.97	0.54	0.04	0.06	0.008
Effect^1^	*GATA2*	*ATGL*	*PLN1*	*CGI-58*	*ACADL*	*NPY*	*NPYR2*	*LPL*
Diet								
HC	0.85	0.65	0.91^a^	0.59	1.58	1.02	0.56	1.33
HF	0.87	0.49	1.09^a^	0.56	1.54	1.01	0.51	1.33
HP	0.73	0.51	0.68^b^	0.50	0.94	1.09	0.44	0.98
SEM	0.05	0.08	0.06	0.08	0.21	0.07	0.05	0.12
*P*-value	0.12	0.34	0.0001	0.71	0.07	0.66	0.19	0.05
Treatment								
Fed	0.86	0.77^a^	0.98^a^	0.84^a^	1.03^b^	0.99^b^	0.79^a^	1.19
Fasted	0.78	0.51^ab^	0.93^ab^	0.37^b^	1.82^a^	1.33^a^	0.39^b^	1.13
Refed	0.81	0.37^b^	0.77^b^	0.45^b^	1.21^ab^	0.81^b^	0.33^b^	1.32
SEM	0.05	0.08	0.06	0.08	0.22	0.07	0.05	0.12
*P*-value	0.57	0.0058	0.04	0.0001	0.04	0.0001	0.0001	0.49
D × T	0.30	0.01	0.87	0.17	0.51	0.54	0.04	0.19

^1^Values represent least squares means and pooled standard errors of the means with associated *P*-values for each effect. D × T: interaction between diet (D) and treatment (T). Different superscipts within an effect for each gene are significantly different at *P* < 0.05, Tukey’s test. Abbreviations: 1-acylglycerol-3-phosphate O-acyltransferase 9: *AGPAT9*; Fatty acid binding protein 4: *FABP4*; CCAAT/enhancer-binding protein alpha and beta: *C/EBPα* and *C/EBPβ*, respectively; Krüppel-like factor 7: *KLF7*; Peroxisome proliferator-activated receptor gamma: *PPARγ*; Monoglyceride lipase: *MGLL*; Sterol regulatory element-binding transcription factor 1: *SREBP1*; GATA-binding protein 2: *GATA2*; Adipose triglyceride lipase: *ATGL*; Perilipin 1: *PLN1*; Comparative gene identification-58: *CGI-58*; Acyl-CoA dehydrogenase, long chain: *ACADL*; Neuropeptide Y: *NPY*; *NPY* receptor 2: *NPYR2*; Lipoprotein lipase: *LPL*

**Table 7:** Abdominal fat mRNA abundance at 4 days post-hatch

Effect^1^	*AGPAT9*	*FABP4*	*C/EBPα*	*C/EBPβ*	*KLF7*	*PPARγ*	*MGLL*	*SREBP1*
Diet								
HC	0.39	0.74	0.36	2.07^a^	1.62^ab^	0.38	4.27^a^	0.43
HF	0.34	0.75	0.36	1.50^ab^	1.63^a^	0.41	3.33^ab^	0.43
HP	0.34	0.71	0.40	1.10^b^	1.33^b^	0.42	2.86^b^	0.38
SEM	0.03	0.05	0.03	0.17	0.08	0.03	0.38	0.03
*P*-value	0.43	0.90	0.56	0.0006	0.02	0.72	0.03	0.15
Treatment								
Fed	0.42^a^	0.69	0.50^a^	1.21^b^	1.32^b^	0.55^a^	4.16^a^	0.64^a^
Fasted	0.35^ab^	0.79	0.27^c^	1.92^a^	1.91^a^	0.27^c^	5.30^a^	0.36^b^
Refed	0.30^b^	0.72	0.36^b^	1.48^ab^	1.32^b^	0.41^b^	1.03^b^	0.26^c^
SEM	0.03	0.05	0.02	0.16	0.08	0.03	0.38	0.03
*P*-value	0.02	0.30	0.0001	0.01	0.0001	0.0001	0.0001	0.0001
D × T	1.00	0.41	0.37	0.46	0.18	0.16	0.11	0.16
Effect^1^	*GATA2*	*ATGL*	*PLN1*	*CGI-58*	*ACADL*	*NPY*	*NPYR2*	*LPL*
Diet								
HC	0.77	1.42	0.78	0.55	0.65^ab^	0.73	0.30	1.51
HF	0.88	0.74	0.56	0.74	0.55^b^	1.02	0.30	1.55
HP	0.67	0.50	0.68	0.58	0.86^a^	1.03	0.31	1.60
SEM	0.07	0.41	0.13	0.07	0.09	0.14	0.04	0.21
*P*-value	0.14	0.25	0.47	0.18	0.04	0.24	1.00	0.86
Treatment								
Fed	0.80	0.60	0.58	0.63	0.67	1.49^a^	0.33^ab^	1.67
Fasted	0.78	0.66	0.74	0.57	0.72	0.84^b^	0.36^a^	1.52
Refed	0.74	1.41	0.71	0.67	0.67	0.44^b^	0.21^c^	1.56
SEM	0.08	0.41	0.12	0.07	0.09	0.15	0.04	0.22
*P*-value	0.83	0.29	0.63	0.68	0.89	0.0005	0.04	0.89
D × T	0.97	0.18	0.24	0.02	0.12	0.48	0.03	0.66

^1^Values represent least squares means and pooled standard errors of the means with associated *P*-values for each effect. D × T: interaction between diet (D) and treatment (T). Different superscipts within an effect for each gene are significantly different at *P* < 0.05, Tukey’s test. Abbreviations: 1-acylglycerol-3-phosphate O-acyltransferase 9: *AGPAT9*; Fatty acid binding protein 4: *FABP4*; CCAAT/enhancer-binding protein alpha and beta: *C/EBPα* and *C/EBPβ*, respectively; Krüppel-like factor 7: *KLF7*; Peroxisome proliferator-activated receptor gamma: *PPARγ*; Monoglyceride lipase: *MGLL*; Sterol regulatory element-binding transcription factor 1: *SREBP1*; GATA-binding protein 2: *GATA2*; Adipose triglyceride lipase: *ATGL*; Perilipin 1: *PLN1*; Comparative gene identification-58: *CGI-58*; Acyl-CoA dehydrogenase, long chain: *ACADL*; Neuropeptide Y: *NPY*; *NPY* receptor 2: *NPYR2*; Lipoprotein lipase: *LPL*

There were also main effects of diet and feeding condition. Expression of fatty acid binding protein 4 (*FABP4*) was greater in chicks that consumed the HC than the HP diet (*P* = 0.02; Table 5). Expression of 1-acylgylcerol-3-phosphate O-acetyltransferase 9 (*AGPAT9*), peroxisome proliferator-activated receptor gamma (*PPARγ*), and sterol regulatory element-binding transcription factor 1 (*SREBP1*) was greater in the subcutaneous adipose tissue of fed than fasted or refed chicks (*P* < 0.05). *C/EBPβ* mRNA was greater in fasted than fed or refed chicks (*P* < 0.05), while Krüppel-like factor 7 (*KLF7*) was greatest in fasted, intermediate in fed, and lowest in the subcutaneous fat of refed chicks (*P* < 0.05).

### mRNA abundance in clavicular adipose tissue

**Fig. 5:**
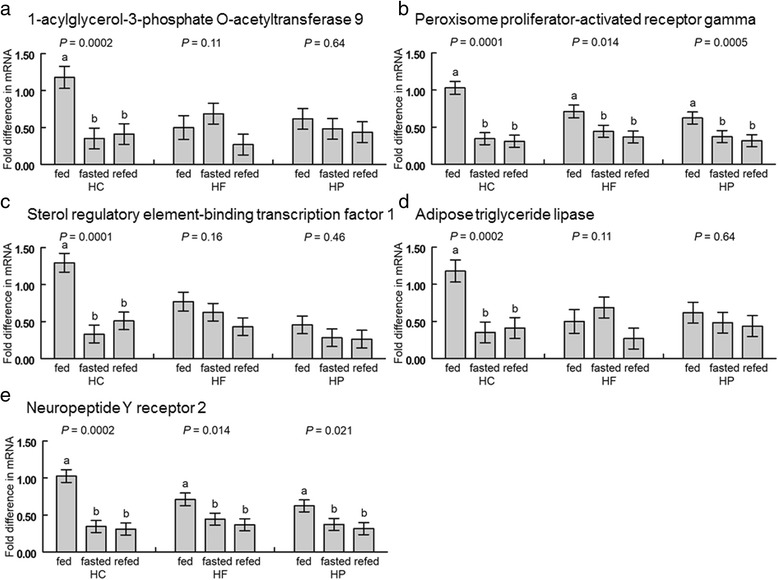
Interactions of diet and feeding treatment on mRNA abundance of **a**) 1-acylglycerol-3-phosphate O-acetyltransferase 9, **b**) Peroxisome proliferator-activated receptor gamma, **c**) Sterol regulatory element-binding transcrption factor 1, **d**) Adipose triglyceride lipase, and **e**) Neuropeptide Y receptor 2 in clavicular fat. Chicks fed one of three diets (HC: high-carbohydrate; HF: high-fat; HP: high-protein) were either continuously fed (fed), fasted for 3 h (fasted) or fasted and refed for 1 h (refed) with *n* =10 per group. Values represent least squares means ± SEM. The *P*-values for the main effect of treatment are displayed above the bars for each dietary group. Different letters within a dietary group indicate a significant difference at *P* < 0.05; Tukey’s test

In clavicular fat, *AGPAT9*, *PPARγ*, *SREBP1*, adipose triglyceride lipase (*ATGL*), and *NPYR2* mRNA quantities were affected by the interaction of feeding treatment and diet (Fig. 5). Expression of *AGPAT9* (Fig. 5a), *SREBP1* (Fig. 5c), and *ATGL* (Fig. 5d) showed similar expression patterns, where in chicks that consumed HC but not the other two diets, mRNA was greater in fed than fasted or refed chicks (*P* < 0.05). The quantities of *PPARγ* (Fig. 5b) and *NPYR2* (Fig. 5e) mRNA showed similar responses, where in all three dietary groups there was greater expression in fed vs. fasted or refed chicks with a greater difference in chicks that consumed the HC diet (*P* < 0.05).

There were multiple main effects of diet and feeding state in clavicular fat. Perilipin1 (*PLIN1*), *FABP4*, and monoglyceride lipase (*MGLL*) mRNAs were greater in the clavicular fat of chicks that consumed the HC and HF than HP diet (P < 0.05; Table 6). Expression of *FABP4* and *PLIN1* mRNA was also greater in fed than refed chicks (*P* < 0.05), while abundance of *C/EBPβ* and acyl-CoA dehydrogenase long chain (*ACADL*) mRNA was greater in fasted than fed chicks (*P* < 0.05). There was greater expression of comparative gene identification-58 (*CGI-58*) in fed chicks than fasted or refed chicks (*P* < 0.05). The expression of Krüppel-like factor 7 (*KLF7*) and *NPY* mRNA was greater in fasted than fed or refed chicks (*P* < 0.05).

### mRNA abundance in abdominal adipose tissue

**Fig. 6:**
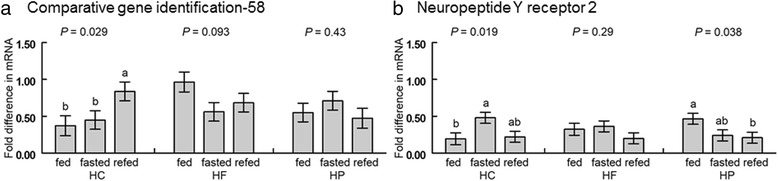
Interactions of diet and feeding treatment on mRNA abundance of **a**) Comparative gene identification-58 and **b**) Neuropeptide Y receptor 2 in abdominal fat. Chicks fed one of three diets (HC: high-carbohydrate; HF: high-fat; HP: high-protein) were either continuously fed (fed), fasted for 3 h (fasted) or fasted and refed for 1 h (refed) with *n* =10 chicks per group. Values represent least squares means ± SEM. The *P*-values for the main effect of treatment are displayed above the bars for each dietary group. Different letters within a dietary group indicate a significant difference at *P* < 0.05; Tukey’s test

The *CGI-58* and *NPYR2* mRNA quantities were affected by the interaction of diet and feeding treatment in abdominal fat (Fig. 6). In chicks that consumed the HC diet, expression of CGI-58 was greater in refed than fasted or fed chicks, while fasting and refeeding had no effects on expression in chicks that consumed the HP or HF diet (*P* < 0.05; Fig. 6a). In chicks that ate the HC diet, *NPYR2* mRNA was greater in fasted than fed chicks, and for chicks that ate the HP diet, expression was greater in the abdominal fat of fed than refed chicks (*P* < 0.05; Fig. 6b).

Similar to the other depots, there were multiple main effects in abdominal fat. The abundance of *C/EBPβ* and *MGLL* mRNA was greater in the HC than HP-fed chicks (*P* < 0.05; Table 7). Expression of *KLF7* mRNA was greater in HF than HP-fed chicks, whereas *ACADL* was greater in HP than HF-fed chicks (*P* < 0.05). There were also many main effects of feeding treatment on mRNA abundance in abdominal fat. Expression of *AGPAT9* was greater in fed than refed chicks (*P* < 0.05). Expression of *C/EBPα* was greatest in fed, lowest in fasted, and intermediate in refed chicks, while *C/EBPβ* was only greater in fasted than fed chicks (*P* < 0.05). The abundance of *KLF7* mRNA was greater in fasted than fed or refed chicks (*P* < 0.05). Expression of *PPARγ* was greatest in fed, intermediate in refed, and lowest in the abdominal fat of fasted chicks (*P* < 0.05). Expression of *MGLL* was greater in fed and fasted than refed chicks (*P* < 0.05). The abundance of *SREBP1* mRNA was greatest in fed, intermediate in fasted, and lowest in refed chicks and *NPY* was greater in fed than fasted or refed chicks (*P* < 0.05).

## Discussion

The current obesity epidemic and projected increases in the prevalence of obesity worldwide necessitate the development of novel research models that provide insights on the pathogenesis of obesity at the cellular and molecular level. This research revealed that not only do different anatomical regions of fat develop differently in chicks, but that dietary fat and protein quantity dramatically influence adipocyte development and lipid remodeling. Three hours of fasting was sufficient to induce a rise in plasma non-esterified fatty acids (NEFAs), however the effect was diet-dependent, and gene expression changes were detected in a diet and adipose tissue depot-dependent manner, further illustrating the complex relationship between diet composition, nutritional state, and anatomical depot-specific physiology. These findings highlight the importance of understanding metabolic differences among subcutaneous and visceral adipose tissue depots and the utility of chick as a model to explore the molecular basis for such differences in humans.

At 4 days post-hatch, there was less adipose tissue deposition in chicks that consumed the HP diet. Feeding more dietary protein (from 18 to 28%) for the first week post-hatch did not affect body fat content in 7 day-old broiler chicks [10], although abdominal fat content was decreased when dietary protein was increased at a later age [11, 12]. In rats, both long term (6 months) or short term (3 weeks) feeding of a HP diet reduced total white adipose tissue (WAT) weights [13, 14]. This may be partially explained by the reduced energy intake from consuming the HP diet, as protein induces greater sensory-specific satiety compared with carbohydrates [15, 16]. However, based on our previous publications, the HP diet does not seem to have a satiating effect on food intake in chicks [5, 17], although the protein content of the HP diet used in the present study was lower as compared to our previous studies (25 vs. 30% CP), and there was no difference in food intake between the HP and other diets during the first 4 days post-hatch [18].

Differences in adipose tissue weight can be partly explained by the gene expression results. In the adipose tissue of chicks consuming the HP diet, there was reduced expression of *FABP4* (subcutaneous and clavicular), *SREBP1* (clavicular) and *C/EBPβ* (abdominal) in comparison with the HC diet, and *FABP4* (clavicular) and *KLF7* (abdominal) compared with the HF diet. The SREBP1 and C/EBPβ are key transcription factors during the early stages of adipogenesis that coordinate the transcriptional regulation of a variety of adipocyte metabolism-associated genes [19–21]. The biological function of FABP4 involves the binding and transport of fatty acids from cell membranes into adipocytes [22]. The KLF7 is a member of the Krüppel-like transcription factor family that promotes chicken preadipocyte proliferation but inhibits its differentiation [23]. Collectively, these results indicate that there might be reduced rates of preadipocyte proliferation and differentiation and associated fatty acid incorporation into triacylglycerols in adipose tissue from chicks fed the HP diet, resulting in less adipose tissue deposition in a depot-specific manner. That adipocyte size and size distribution were similar between HP and HC diet-fed chicks also supports that in the HP diet-fed chicks the reduced weight of the adipose tissue was due to reduced numbers of adipocytes, possibly from less adipogenesis, compared with HC diet-fed chicks.

In mice more than 6 weeks old, 60 days of feeding a HF diet revealed that visceral fat expanded predominantly by adipocyte hypertrophy, whereas subcutaneous fat expanded by adipocyte hyperplasia [4]. Increased intra-abdominal/visceral fat is associated with a greater risk of developing metabolic diseases, whereas increased subcutaneous fat in the thighs and hips represents little or no risk [24]. A relative lack of progenitor cell activity may be the reason why adipose depots such as visceral accumulate hypertrophic, dysfunctional adipocytes and are consequently associated with a higher risk of metabolic diseases [4]. Our results revealed that consumption of the HF diet was associated with larger adipocytes. Consistent with our previous research [6], fat depot weight and adipocyte area and diameter were greater in subcutaneous and clavicular than abdominal fat at day 4 post-hatch. The high percentage of small adipocytes (>91% were 10 to 25 μm) in the abdominal fat along with more of what appear to be preadipocyte clusters may suggest that a combination of hyperplasia and hypertrophy contribute to the adipose tissue development of abdominal fat, the depot that develops at a more rapid rate post-hatch than other depots in broiler chickens [6].

Expression of genes involved in the early stage of adipogenesis, the commitment of mesenchymal stem cells to form new adipocytes, was downregulated in the abdominal fat of broiler chickens after 5 h of fasting [25]. In the present study, fasting downregulated the mRNA abundance of *PPARγ*, *SREBP1*, and *C/EBPα* (except in the clavicular fat) and upregulated *C/EBPβ* and *KLF7* mRNA in all three adipose tissue depots. Similarly, 4 h of fasting decreased adipose tissue *PPARγ* and *SREBP1* mRNA in 13 day-old chicks [26]. These effects are also consistent with effects of 4 and 8 h of fasting on *PPARγ* and *SREBP1* mRNA in the adipose tissue of rats [27, 28]. In rodents, PPARγ and SREBP1 are considered to be lipogenic transcription factors in the adipose tissue, as de novo lipogenesis occurs at a greater rate in the adipose tissue of rodents compared to humans or birds. The PPARγ is able to activate glucose transporter 4 expression, indicative of increased fatty acid synthesis from glucose [29]. SREBP1 regulates the expression of key genes involved in lipid and glucose metabolism, such as *FAS* and acetyl-coenzyme A carboxylase [30, 31]. However, unlike many mammals in which adipocytes are the main site of *de novo* lipogenesis, *de novo* lipogenesis is considered to take place almost exclusively in the liver of avian species, similar to humans [32]. Therefore, decreased *PPARγ* and *SREBP1* mRNA expression in chicken adipose tissue after fasting does not necessarily suggest reduced lipogenesis in response to fasting, but may reflect a downregulation of triacylglycerol synthesis in the absence of available substrate.

Except for its roles in lipogenesis, the activation of PPARγ also promotes terminal differentiation, which is achieved through the induction of a variety of differentiation-dependent genes that are crucial for fatty acid uptake and storage, such as *FABP4*, *LPL* and others [21]. In addition to promoting adipocyte differentiation, PPARγ also plays an important role in adipocyte hypertrophy in the adipose tissue, upregulating expression of enzymes such as LPL in the adipocyte [33]. As a critical adipogenic regulator, C/EBPα also induces the expression of differentiation-associated factors [21].

Although *PPARγ* and *SREBP1* had similar expression patterns in each adipose depot in response to fasting, there were some depot-dependent changes. Fasting decreased *AGPAT9* mRNA in subcutaneous fat, decreased *NPY* in abdominal fat, while it increased *NPY* expression in clavicular fat, and decreased *NPYR2* in clavicular and subcutaneous fat. The *AGPAT9* encodes an enzyme that catalyzes the initial step of *de novo* triacylglycerol synthesis [34]. Sympathetic neuron-derived NPY and activation of its receptor NPYR2 promote adipocyte proliferation and differentiation [35], and knockout of *NPYR2* resulted in reduced body weight gain and adiposity [36]. Increased lipolysis during fasting is expected to be accompanied by a corresponding reduction in triacylglycerol synthesis/lipogenesis. Collectively, these changes indicate that transcriptional regulation of adipogenesis-associated factors is altered in response to fasting in order to down-regulate preadipocyte differentiation and decrease adipocyte hypertrophy in a depot-dependent manner.

In rats, circulating NEFAs increased after 4 h of fasting and peaked at 8 h, however, *ATGL* mRNA was increased in retroperitoneal WAT after 24 but not 8 h of fasting [28]. Thus, the rapid increase in circulating NEFAs under fasting conditions is most likely explained by an upregulation of enzyme activity, occurring prior to changes induced at the transcriptional or translational level [28]. Adipose triglyceride lipase and hormone sensitive lipase (HSL) are the major enzymes involved in triacylglycerol catabolism, accounting for almost all hydrolase activity in murine WAT [37]. However, no HSL orthologue has been identified in the chicken genome [38], suggesting an even more important role of ATGL in lipolysis in chickens. In the present study, *ATGL* mRNA was not upregulated after 3 h fasting in any of the three adipose depots, indicating that lipolysis might be promoted by an upregulation of the enzyme at the protein level, similar to mammalian studies, although it should be noted that 3 h of fasting increased plasma NEFA concentrations only in chicks that consumed the HC and HP diets.

Under basal conditions, PLN1 may inhibit lipolysis by blocking the binding of lipase to triacylglycerols and/or by sequestering CGI-58 [39]. In the activated state, phosphorylation of PLN1 results in the release of CGI-58, which can then activate ATGL [40]. Therefore, further research is needed to evaluate how fasting affects phosphorylated PLN1 levels in the WAT of chickens. MGLL liberates the last fatty acid from glycerol; *MGLL* was not upregulated after fasting in any adipose tissue depot. Fasting increased *NPY* and *ACADL* mRNA and decreased *CGI-58* mRNA expression in the clavicular fat of chicks. ACADL is responsible for the first step of the β-oxidation of long chain fatty acids in the mitochondria [41]. The differential expression of these genes might indicate that the clavicular adipose tissue has a different metabolic response to fasting than subcutaneous and abdominal fat.

According to Nguyen et al. (2014), there was a greater sympathetic drive, triggered by food deprivation, to subcutaneous (inguinal) WAT than visceral (mesenteric) WAT depots, with increased sympathetic nervous system drive only being observed in the subcutaneous (inguinal) WAT after the lipolytic stimulus of food deprivation in Siberian hamsters [42]. In general, sympathetic stimulation promotes lipolysis in WAT [43]. Thus, there might be a proportionally greater increase in lipolysis in the subcutaneous fat than the other two depots.

Similar to fasting, a cascade of events occurs during refeeding after fasting to allow tissues such as adipose tissue to adapt metabolically in order to restore nutritional homeostasis. In the WAT of rats, 3 h of refeeding was sufficient to restore the mRNA expression of *PPARγ* and *SREBP1* to control levels after 8 h of fasting, however, this time did not allow for the recovery of *FAS* [28]. In the present study, fasting increased plasma NEFA concentrations in chicks fed the HC and HP diets, and 1 h of refeeding restored the plasma NEFA concentrations to the fed state. However, 1 h of refeeding was not sufficient for restoring the fed-state levels of mRNA expression of some adipogenesis-associated factors, such as *PPARγ* and *SREBP1*.

## Conclusions

In conclusion, reduced adipose tissue deposition in chicks fed a HP diet might be explained by decreased rates of adipogenesis and associated fatty acid incorporation into triacylglycerol synthesis. Conversely, chicks that consumed the HF diet had heavier fat pads and larger adipocytes, likely as a result of greater rates of adipocyte hypertrophy. In response to fasting, transcriptional regulation of adipogenesis-associated factors is induced, the net effect likely being an inhibition of preadipocyte differentiation and adipocyte hypertrophy, in a depot-specific manner. The lipolytic response to 3 h of fasting in chicks that consumed the HP diet also supports that the balance between adipogenesis/hypertrophy and lipid catabolism is shifted to favor a reduced rate of fat deposition. These results may provide implications for understanding how dietary macronutrients affect the development of different adipose tissue depots during the early life period and mechanisms underlying depot-dependent differences in metabolism across species.

## Abbreviations

*ACADL*: Acyl-CoA dehydrogenase long chain
*AGPAT9*: 1-acylglycerol-3-phosphate O-acetyltransferase 9
*ATGL*: Adipose triglyceride lipase
*C/EBPα*: CCAAT/enhancer-binding protein alpha
*C/EBPβ*: CCAAT/enhancer-binding protein beta
*CGI-58*: Comparative gene identification-58
CP: crude protein
*FABP4*: Fatty acid binding protein 4
*FAS*: Fatty acid synthase
*GATA2*: GATA binding protein 2
HC: High carbohydrate
HF: High fat
HP: High protein
*KLF7*: Krüppel-like factor 7
*LPL*: Lipoprotein lipase
*MGLL*: Monoglyceride lipase
NEFA: Non-esterified fatty acid
*NPY*: Neuropeptide Y
*NPYR2*: NPY receptor 2
*PLN1*: Perilipin 1
*PPARγ*: Peroxisome proliferator-activated receptor gamma
*SREBP1*: Sterol regulatory element-binding transcription factor 1
WAT: White adipose tissue
